# Evaluation of Risk Factors in Patients With Chronic Daily Headache and Medication‐Overuse Headache

**DOI:** 10.1002/brb3.70407

**Published:** 2025-03-13

**Authors:** Gülcan Taşçatan, Hüseyin Tuğrul Atasoy, Esra Acıman Demirel, Vildan Çakır Kardeş, Mustafa Açıkgöz, Ulufer Çelebi, Bilge Piri Çınar, Bilgehan Açıkgöz, Aynur Özge

**Affiliations:** ^1^ Zonguldak Bülent Ecevit University, Mersin University Yenişehir/Mersin Türkiye; ^2^ Zonguldak Bulent Ecevit University Faculty of Medicine Department of Neurology Turkey; ^3^ Zonguldak Bulent Ecevit University Faculty of Medicine Department of Mental Health and Diseases Turkey; ^4^ Samsun University Faculty of Medicine Department of Neurology Turkey; ^5^ Zonguldak Bulent Ecevit University Faculty of Medicine Department of Public Health Turkey; ^6^ Mersin University Faculty of Medicine Department of Neurology Turkey

**Keywords:** addiction, chronic daily headache, medication‐overuse headache, migraine, tension‐type headache

## Abstract

**Purpose:**

The aim of this study is to compare the quality of life, daily physical activity level and perception of illness in patients with chronic daily headache, medication‐overuse headache, episodic migraine, and episodic tension‐type headache across disease subgroups with respect to possible risk factors such as alcohol/caffeine/nicotine use, eating attitude, which may trigger chronic headache and medication overuse.

**Materials and Methods:**

VAS, International Physical Activity Questionnaire (Short) forms, SF‐36 Quality of Life Scale, Illness Perception Questionnaire, Beck Anxiety Inventory, Beck Depression Inventory, Eating Disorder Examination Questionnaire, Eating Attitude Test, SCID‐I were applied to the patients.

**Results:**

Disease type mean score and psychological causes mean score were found to be the highest in the chronic tension type headache (CTTH) group among tension type headache (TTH) subgroups. Among the subgroups of migraine patients, the mean score of risk‐related causes was found to be the highest in chronic migraine with medication overuse (MOH‐M). The mean score of psychological causes was found to be significantly higher in patients with CTTH with medication overuse (TTH‐MOH) compared to patients with MOH‐M. Worsening of body pain was significant in CDH‐M and MOH‐M, whereas worsening in physical role difficulty, emotional well‐being, general health perception was significant in CTTH and TTH‐MOH.

**Conclusions:**

In the TTH group, BMI was found to be statistically significantly higher compared to the migraine group. Similarly, in the TTH‐MOH group, BMI was higher than in the MOH‐M group. This finding suggests that the relationship between TTH and obesity warrants further attention. Our study also showed that patients with TTH‐MOH experience greater difficulties in daily life and work compared to those in the MOH‐M group. Additionally, TTH‐MOH patients are more likely to attribute the cause of their current headaches to psychogenic factors, in comparison to MOH‐M patients. Findings suggest that psychosocial factors have a greater influence on the chronicity of the disease and the emergence of medication overuse in the TTH group compared to the migraine group.

## Graphical abstract

Headache Types and Prevalence–Representation of migraine and tension‐type headache; the subgroups with episodic, chronic daily headache, and medication‐overuse headache. Key Risk Factors–Overuse of medication, psychosocial stress, high BMI, lifestyle factors (alcohol, caffeine, nicotine use). Psychological & Quality of Life Impact–Links between headache severity, depression, anxiety, and decreased physical role function. Statistical Findings–Comparative risk scores among headache subtypes, emphasizing differences in BMI, pain perception, and psychiatric comorbidities

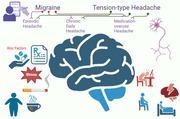

## 1 Introduction

Headache complaints rank first among the reasons for seeking neurology clinic consultation, as it significantly affects quality of life and imposes limitations (İdiman et al. 2015). Currently, 46% of the world population actively experiences headaches, with a prevalence of 42% for tension‐type headache, 11% for migraine, and 3% for chronic daily headache (Stovner et al. 2007). Headache occurring for more 15 days a month for at least 3 months is classified as chronic daily headache (Murinova and Krashin 2015). Primary headaches, such as chronic migraine or chronic tension‐type headache as well as secondary headaches like chronic cluster headache or new daily persistent headache, can lead to chronic daily headache (Murinova and Krashin 2015). The prevalence of medication‐overuse headache, which is often caused by the excessive use of medications used to treat tension‐type headache or migraine, is around 1%–2% (Kristoffersen and Lundqvist 2014; Atasoy et al. 2005).

Risk factors, such as initial headache frequency of 7–14 days per month, sedentary lifestyle, being under 50 years, high body mass index (BMI), smoking, low socioeconomic status, stress, anxiety, and mood disorders, play a role in the development of medication‐overuse headache (Atasoy et al. 2005; Autret et al. 2010; Schwedt et al. 2018). Having individuals with medication overuse or substance abuse in the family and daily caffeine consumption exceeding 200 mg/day also increase the risk of medication‐overuse headache (Ertaş and Başağrısı 2018).

This study evaluated the quality of life, daily physical activity level, illness perception, eating behaviors, and consumption of alcohol, caffeine, and nicotine that may contribute to the chronicity of headaches and excessive medication intake in patient groups with episodic migraine, episodic tension‐type headache, chronic daily headache, and medication‐overuse headache and compared the groups on the basis of these parameters.

## 2 Materials and Methods

### 2.1 Study Population

**FIGURE 1:**
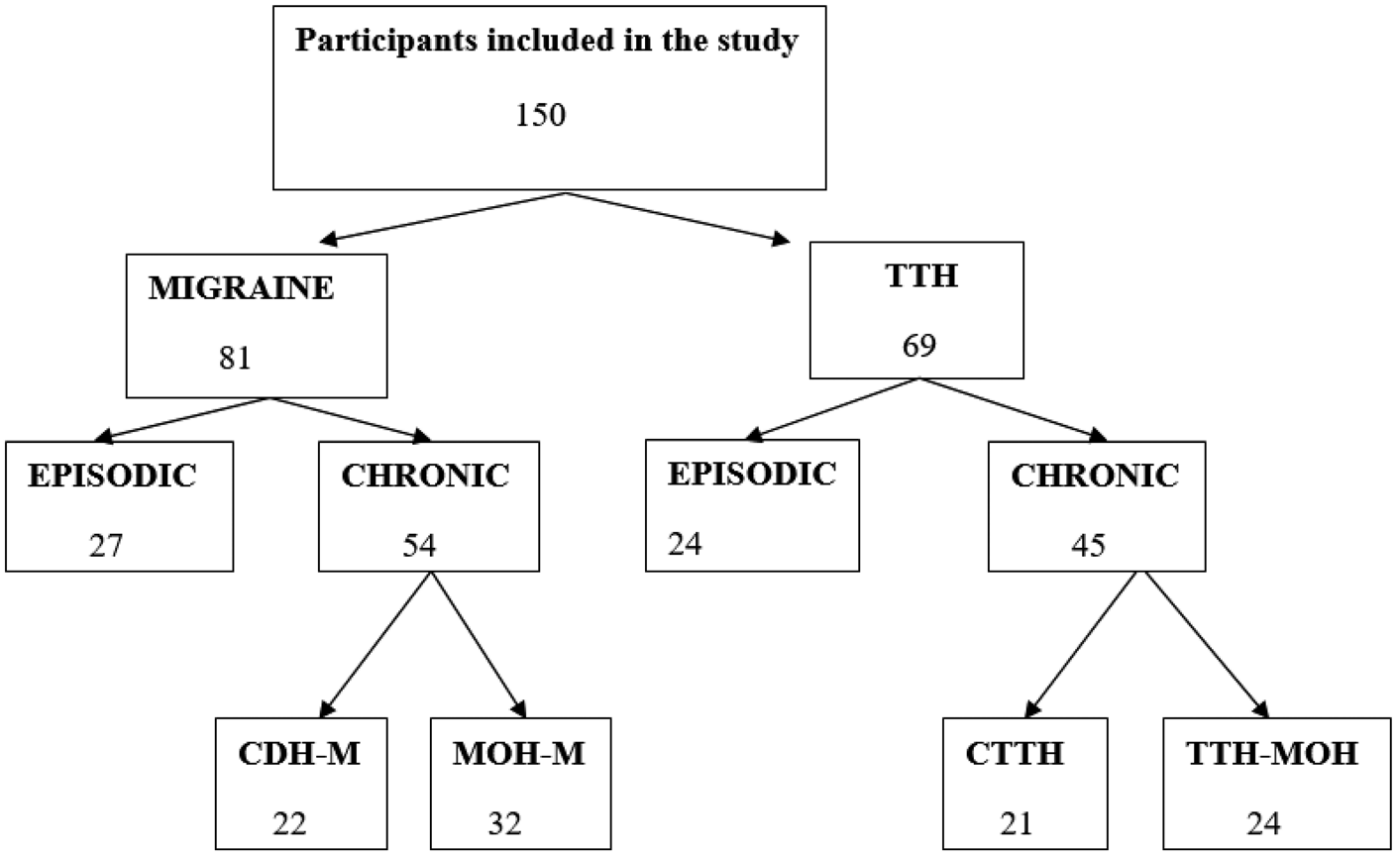
Study Group. TTH: Tension‐type headache. CDH‐M: Chronic daily headache with migraine. CTTH: Chronic tension‐type headache. MOH‐M: Medication‐overuse headache with migraine. TTH‐MOH: Tension‐type headache with medication‐overuse.

History and clinical examinations of patients who applied to the Neurology Polyclinic of Zonguldak Bülent Ecevit University Health Application and Research Center with complaints of headache between January 1, 2020, and December 1, 2020, were performed to determine headache subtypes. Exclusion criteria were defined as having a diagnosis of intracranial mass, being under 18 years of age, lacking the cognitive ability to complete the scales, and refusal to participate in the study. Patients were provided with detailed information about the study, and signed informed consent forms were obtained from those who agreed to participate in the study. Patients who agreed to participate in the study were divided into two groups according to the criteria of The International Classification of Headache Disorders 3rd edition (ICHD‐3): migraine and tension‐type headache groups. The subgroups with episodic, chronic daily headache, and medication‐overuse headache were compared within these two groups (Figure 1). In determining the subgroups, the ICHD‐3 criteria were also taken into account and the subgroups are mutually exclusive. Ethical approval was obtained from Zonguldak Bülent Ecevit University Clinical Research Ethics Committee on February 19, 2020 (Meeting no: 2020/04). Tof the included in the study according to headache subtypes is shown in Tab

### 2.2 Study Design

The participants included in the study underwent a neurological examination, and a sociodemographic data form was completed, which included inquiries about the amounts of alcohol, caffeine, and nicotine consumption. Headache frequency and duration were also recorded during clinical interviews with the patient. Visual analog scale (VAS) was used to assess pain intensity. To identify accompanying psychiatric conditions such as depression, anxiety, and eating disorders, the Beck Depression Inventory (BDI) and Beck Anxiety Inventory (BAI), Eating Attitudes Test‐40 (EAT‐40), and Eating Disorder Examination Questionnaire (EDE‐Q) were filled out under the supervision of a physician. To assess the quality of life, the Short Form Health Survey (SF‐36) scale was utilized, and the International Physical Activity Questionnaire was used for the classification of physical activity. Illness Perception Questionnaire (IPQ) was also completed in the presence of a physician. The language of the scales used in the study is Turkish, and patients with vision and writing problems were supported in filling out the scales. Additionally, to better assess the diagnosis of accompanying psychiatric disorders, a psychiatric interview was conducted with patients using the Structured Clinical Interview for DSM‐IV (SCID‐I) by a psychiatrist.

### 2.3 Statistical Analysis

The data were transferred to a digital environment and analyzed using the RStudio 1.4 program. The normality of the data was assessed using the Shapiro–Wilk test, and descriptive statistics were presented in the form of number (*n*), percentage (%), mean ± standard deviation (mean ± SD). Pearson's Chi‐square test, Fisher's exact test, and Yates‐corrected Chi‐square test were used to analyze categorical data. Student's *t*‐test, Kruskal‐Wallis variance analysis, and Mann–Whitney *U* test were used in the analysis of numerical variables. *p* < 0.05 was considered statistically significant in all analyses.

## 3 Results

### 3.1 Sociodemographic Data

Sociodemographic data of migraine and TTH patients are shown in Table 1. The mean age of patients diagnosed with TTH was found to be higher than that of patients diagnosed with migraine, and this difference was statistically significant (*p* = 0.001). On comparing the disease duration of patients diagnosed with migraine and TTH, it was found that disease duration was longer in the migraine group compared to the TTH group, and the difference was statistically significant (128 ± 121.1, 90 ± 14.4, *p* = 0.042, respectively). VAS scores were significantly higher in the migraine group (*p* = 0.001). The duration of attacks in the migraine group was significantly longer compared to the TTH group (*p* = 0.004) (Table 2). When the migraine group was evaluated, the frequency of headaches in the CDH‐M and MOH‐M groups was significantly higher compared to the episodic group (*p* < 0.001). In the TTH group, disease duration, VAS score, headache frequency, and headache duration were significantly higher in the CTTH and TTH‐MOH groups compared to the episodic TTH subgroup (Table 3). When patients diagnosed with episodic migraine and episodic TTH were compared, it was found that patients with episodic TTH had a significantly higher mean age, while patients with episodic migraine had significantly higher mean disease duration, VAS score, and headache duration (Table 4).

**TABLE 1:** Sociodemographic data.

	Migraine (*n* = 81)	TTH (*n* = 69)	*p* value
**Gender** Female, *n* (%) Male, *n* (%)	69 (85.2%) 12 (14.8%)	50 (72.5%) 19 (27.5%)	0.086
**Average age**	38.3 ± 11.5	46.9 ± 15.0	**0.001** ^*^
**Marital status** Married, *n* (%) Single, *n* (%)	59 (72.8%) 22 (27.2%)	57 (82.6%) 12 (17.4%)	0.219
**Profession** Student, *n* (%) Employed, *n* (%) Unemployed, *n* (%)	8 (9.87%) 26 (32.09%) 47 (58.02%)	3 (4.34%) 20 (28.98%) 46 (66.66%)	0.347
**Educational status** University and above, *n* (%)	22 (27.2%)	8 (11.6%)	**0.030** ^*^
**Smoking**, *n* (%)	33 (40.7%)	21 (30.4%)	0.254
**Alcohol use**, *n* (%)	6 (7.4%)	3 (4.3%)	0.508

* *p* < 0.05.

**TABLE 2:** Disease characteristics of migraine and TTH patients.

	Migraine (*n* = 81)	TTH (*n* = 69)	*p* value
**Disease duration** (month)	128 ± 121.1	90 ± 104.4	**0.042** ^*^
**Mean VAS score**	8.1	7.2	**0.001** ^*^
**Frequency of headache in a month** (days/month)	4.76	4.58	0.369
**Average duration of headache during an attack (h)**	4.03	3.60	**0.004** ^*^

* *p* < 0.05.

**TABLE 3:** Comparison of age, disease duration, VAS score, headache frequency and duration of headaches by disease subgroups.

	Migraine (*n* = 81)				TTH (*n* = 69)			
Type	Episodic (*n* = 27)	CDH‐M (*n* = 22)	MOH‐M (*n* = 32)	*p* value	Episodic (*n* = 24)	CTTH (*n* = 21)	TTH‐MOH (*n* = 24)	*p* value
**Age**	38.92 ± 11.14	37.18 ± 12.04	38.65 ± 11.67	0.963	48.45 ± 16.36	42.57 ± 16.36	49.16 ± 13.94	0.488
**Disease duration** (month)	110.55 ± 95.38	104.31 ± 121.20	159.46 ± 136.70	0.295	64.54 ± 100.74	97.90 ± 134.66	109.50 ± 72.28	0.016^*^
**VAS**	7.88 ± 1.55	8.40 ± 1.59	8.09 ± 1.57	0.386	5.87 ± 1.87	7.52 ± 1.32	8.33 ± 0.86	**<** **0.001** ^*^
**Headache frequency** (days/month)	3.25 ± 0.76	15.22 ± 0.42	15.34 ± 1.03	**<** 0.001^*^	3.37 ± 1.24	15.61 ± 0.49	15.41 ± 0.71	**<** **0.001** ^*^
**Duration of headaches** (h)	4.07 ± 0.82	4.04 ± 0.72	4.00 ± 0.76	0.907	3.16 ± 0.86	3.80 ± 1.16	3.87 ± 0.85	**0.019** ^*^

* *p* < 0.05.

**TABLE 4:** Comparison of age, disease duration, VAS score, Headache frequency and duration of headaches in patients with episodic migraine and episodic TTH.

	Episodic migraine (*n* = 27)	Episodic TTH (*n* = 24)	*p* value
**Age**	21.92	30.24	**0.046** ^*^
**Disease duration** (month)	30.96	20.84	**0.014** ^*^
**VAS**	8.00	6.00	**0.002** ^*^
**Headache frequency** (days/month)	3.25	3.37	0.365
**Duration of headaches** (h)	33.02	18.70	**<** **0.001** ^*^

* *p* < 0.05.

The mean VAS score was 8.40 in the CDH‐M group and 7.52 in the CTTH group (*p* = 0.025). The mean headache frequency was 26.31 in the CTTH group and 17.89 in the CDH‐M group, and the difference was statistically significant (*p* = 0.010). The mean age of patients in the TTH‐MOH group was 35.73 years, while the mean age of patients in the MOH‐M group was 23.08 years. The mean age in the migraine group was significantly lower compared to the TTH group (*p* = 0.004).

### 3.2 Daily Living Activities and Habits

When migraine and TTH groups were evaluated in terms of factors (caffeine consumption, tea consumption, exercise duration, physical activity scale, BMI) that could affect headaches, only BMI was found to be statistically significantly higher in the TTH group compared to the migraine group (27.98 vs. 25.55, *p* = 0.011). Body mass index, exercise duration, and physical activity scales were assessed independently. When eating attitudes were evaluated with the EAT‐40 and EDE‐Q (total score, restriction, eating anxiety, body anxiety and weight anxiety) in migraine and TTH groups, no significant difference was found between the groups.

When MOH‐M and TTH‐MOH groups were compared in terms of average exercise duration and physical activity scale values, mean BMI was found to be higher in the TTH‐MOH group (24.78 vs. 33.46, *p* = 0.049). Mean score for the physical role limitations subscale of SF‐36 was significantly higher in the MOH‐M group compared to the TTH‐MOH group (32.20 vs. 23.56, *p* = 0.036). The differences between the groups for the rest of the SF‐36 subscales were not statistically significant.

### 3.3 Disease Perception and Quality of Life

When the eight subgroups evaluated in the SF‐36 were compared between the migraine and TTH groups, no statistically significant difference was found. When the subscales of SF‐36 were evaluated in the TTH group, the episodic group had higher mean scores for physical role limitations, energy, emotional well‐being, bodily pain, and general health perception (Table 5). However, the statistically significant differences found in the “physical role limitations, emotional well‐being, bodily pain, and general health perception” subscales of SF‐36 were due to the high scores in the episodic TTH subgroup.

**TABLE 5:** Comparison of SF‐36 Subscales between TTH subgroups.

	TTH (*n* = 69)			
Type	Episodic (*n* = 24)	CTTH (*n* = 21)	TTH‐MOH (*n* = 24)	*p* value
**Physical function**	73.12 ± 31.37	76.19 ± 22.46	60.41 ± 29.22	0.138
**Physical role difficulty**	52.70 ± 45.10	41.19 ± 42.77	20.83 ± 33.51	**0.028** ^*^
**Emotional role difficulty**	63.87 ± 43.86	53.96 ± 47.69	33.32 ± 42.84	0.062
**Energy/vitality**	56.04 ± 15.60	38.80 ± 19.67	42.08 ± 15.73	**0.002** ^*^
**Emotional well‐being**	66.00 ± 12.31	41.52 ± 19.45	53.33 ± 16.75	**<** **0.001** ^*^
**Social functioning**	59.68 ± 22.71	58.57 ± 24.18	54.89 ± 20.79	0.746
**Bodily pain**	60.93 ± 19.88	40.00 ± 20.29	32.39 ± 14.36	**<** **0.001** ^*^
**General health perception**	60.20 ± 15.49	46.66 ± 19.83	38.75 ± 16.16	**<** **0.001** ^*^

* *p* < 0.05.

**FIGURE 2:**
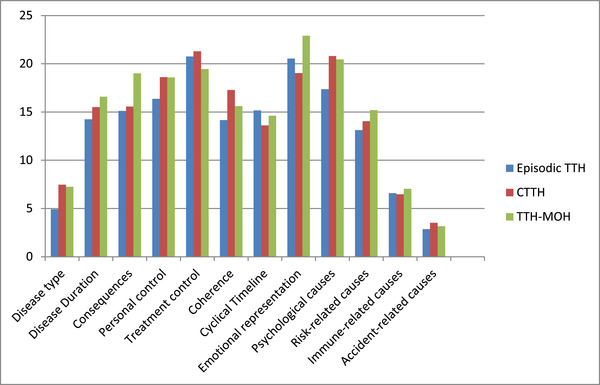
Comparison of Illness Perception Questionnaire subscales between TTH subgroups.

Similarly, the statistically significant difference found in the “disease consequences” subscale of IPQ was due to the high scores in the TTH‐MOH subgroup (Figure 2). CTTH and TTH‐MOH groups should be further evaluated using these two scales with a higher number of patients. A significant difference was found only in the “physical role limitations” scores between the MOH‐M and TTH‐MOH subgroups (Table 6).

**TABLE 6:** Comparison of SF‐36 subscales between the MOH‐M and TTH‐MOH groups.

	MOH‐M (*n* = 32)	TTH‐MOH (*n* = 24)	*p* value
**Physical function**	30.58	25.73	0.269
**Physical role difficulty**	32.20	23.56	**0.036** ^*^
**Emotional role difficulty**	30.95	25.23	0.168
**Energy/vitality**	27.83	29.40	0.720
**Emotional well‐being**	29.59	27.04	0.561
**Social functioning**	26.22	31.54	0.220
**Bodily pain**	28.52	28.48	0.993
**General health perception**	31.30	24.77	0.136

* *p* < 0.05.

**FIGURE 3:**
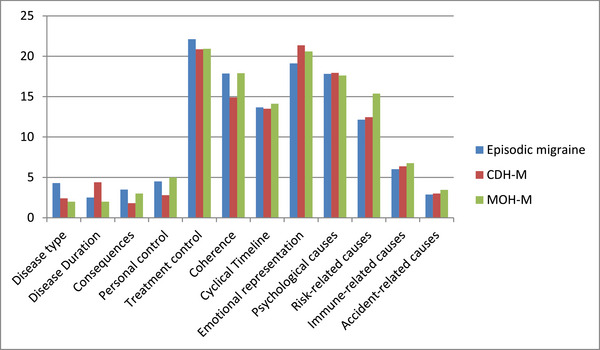
Comparison of Illness Perception Questionnaire subscales between the migraine subgroups.

When the subscales of IPQ were evaluated in the migraine group, the difference observed between the groups in the “risk and causes” subscale was due to the high value in the MOH‐M group (Figure 3). CTTH and MOH‐M groups should be further evaluated using the IPQ with a higher number of patients.

**FIGURE 4:**
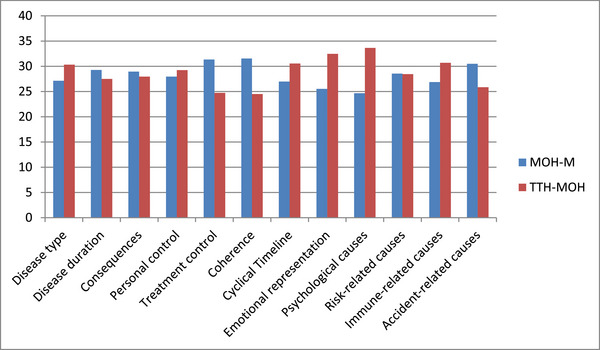
Comparison of IPQ subscales between the MOH‐M and TTH‐MOH groups.

“Consequences” subscale scores of IPQ were significantly higher in the CDH‐M group compared to the CTTH group (*p* = 0.020) (Table 7). However, the mean score of “psychological causes” subscale was found to be higher in the TTH‐MOH group compared to the MOH‐M group (33.63 vs. 24.66, *p* = 0.041) (Figure 4).

**TABLE 7:** Comparison of IPQ subscales between the CDH‐M and CTTH groups.

	CDH‐M (*n* = 22)	CTTH (*n* = 21)	*p* value
**Disease type**	20.20	23.88	0.332
**Disease duration**	22.75	21.21	0.685
**Consequences**	26.32	17.48	**0.020** ^*^
**Personal control**	22.18	21.81	0.922
**Treatment control**	21.39	22.64	0.742
**Coherence**	19.93	24.17	0.265
**Cyclical timeline**	21.61	22.40	0.835
**Emotional representation**	24.16	19.74	0.246
**Psychological causes**	19.45	24.67	0.172
**Risk‐related causes**	20.89	23.17	0.550
**Immune‐related causes**	23.57	0.363	0.911
**Accident‐related causes**	20.50	23.57	0.363

* *p* < 0.05.

### 3.4 Psychogenic Factors and Psychiatric Comorbidity

No statistically significant difference was found in BAI and BDI scores between the migraine and TTH groups (*p* = 0.397, *p* = 0.520, respectively). However, within the TTH group, BAI and BDI scores were significantly higher in the CTTH subgroup compared to the other subgroups (*p* = 0.001, *p* = 0.006, respectively).

**FIGURE 5:**
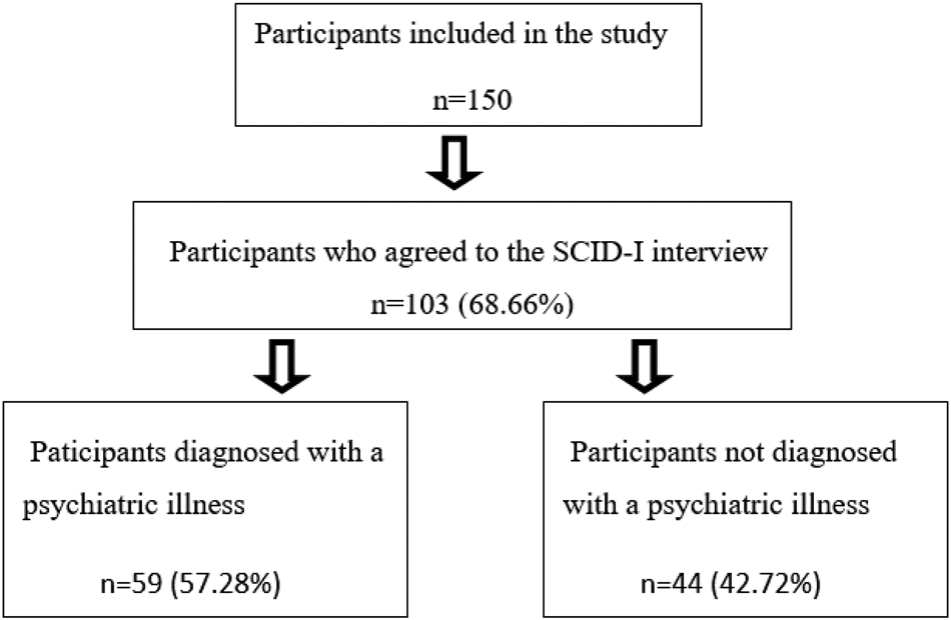
Numerical data on patients participating in the SCID interview.

Patients included in the study were also interviewed by a psychiatrist using the Structured Clinical Interview for DSM‐IV Axis I Disorders‐Clinical Version (SCID‐I) to determine any psychiatric comorbidity. Numerical data related to patients undergoing SCID interviews are presented in Figure 5. Detailed information on diagnoses according to disease subgroups is shown in Tables 8 and 9. When the difference between depressive disorders and anxiety disorders was analyzed, no statistically significant difference was found between the migraine subgroups (*p* = 0.498). Similarly, no statistically significant difference was found between the TTH subgroups (*p* = 0.304).

**TABLE 8:** Psychiatric co‐ diagnoses detected according to SCID interview.

Comorbid psychiatric diagnoses identified	Number of patients (*n*)	Percentage (%)
Depressive disorders	*n* = 35	33.98%
Anxiety disorders	*n* = 37	35.92%
Other (bipolar | disorder, schizophrenia, somatoform disorder)	*n* = 5	4.85%

**TABLE 9:** Distribution of psychiatric diagnoses according to SCID‐I assessment results.

	Episodic migraine (*n* = 16)	CDH‐M (*n* = 16)	MOH‐M (*n* = 21)	Episodic TTH (*n* = 18)	CTTH (*n* = 15)	TTH‐MOH (*n* = 17)
**Dysthymic disorder**	1				1	
**Adjustment disorder with depressive symptoms**	3	2	4	1	4	3
**Mood disorder due to general medical condition**						1
**Major depressive disorder**	2	2	2	1	5	2
**Adjustment disorder with anxiety**			1	1		3
**Anxiety disorder due to general medical condition**			1	1		
**Generalized anxiety disorder**	1	1	1		2	
**Panic disorder**	6	1	2	1		2
**Post‐traumatic stress disorder**	3	1	3	1	1	1
**Obsessive compulsive disorder**	1	2	1		1	1
**Phobias**	1	1	2			
**Bipolar I disorder**		1	1			1
**Schizophrenia**						1
**Somatoform disorder**					1	

While the average age of patients diagnosed with TTH was higher than migraine patients, their education level was lower.

Migraine patients experience headaches for a longer time than TTH patients, and the duration and intensity of pain during the attack are higher.

Among migraine patients, the frequency of pain was higher in CDH‐M and MOH‐M patients than in the episodic group, while in patients diagnosed with TTH, pain frequency as well as pain duration, VAS value, and disease duration were found to be higher in the CTTH and TTH‐MOH group compared to the episodic group.

Since the onset age of patients diagnosed with episodic TTH was older than episodic migraine patients, the duration of the disease was found to be shorter. Pain duration and VAS values of migraine patients were found to be higher than patients diagnosed with TTH.

When TTH patients are evaluated among themselves, it shows that the episodic group has a higher quality of life than other subgroups.

CTTH patients attribute their common pain/symptoms (nausea, fatigue, difficulty sleeping, etc.) to existing headaches and attribute their headaches mostly to psychological causes.

Compared to MOH‐M patients, TTH‐MOH patients experience more difficulties in daily or work life in terms of the difficulties caused by the disease.

Drug overuse in migraine patients is related to the chronicity of pain.

In patients diagnosed with CTTH, the physical, social, financial and psychological effects of the disease are lower than in patients diagnosed with CDH‐M.

Patients diagnosed with TTH‐MOH attribute their illness to psychological causes more than patients diagnosed with MOH‐M.

Anxiety disorders are more common in patients.

## 4 Discussion

### 4.1 Demographic Data

Migraine typically tends to onset in late childhood and adolescence, while TTH tends to onset in the 1920s (Selçuki et al. 2018). In the present study, the higher average age of TTH patients, coupled with the longer duration of the disease in the migraine patient group, supports the previously reported age difference between migraine and TTH. Furthermore, the significantly higher average age of patients diagnosed with TTH‐MOH compared to those diagnosed with MOH‐M also highlights the age difference between the migraine and TTH groups.

In the present study, mean VAS score was significantly higher and the mean duration of headache during an attack was significantly longer in the migraine group compared to the TTH group. In addition, the mean VAS score was higher in CDH‐M patients compared to CTTH patients. Conversely, mean frequency of headaches was higher in CTTH patients compared to CDH‐M patients. When IPQ scores were evaluated for migraine subgroups, it was found that “risk‐related causes” subscale scores were highest in MOH‐M patients. Similarly, “timeline” subscale scores were also highest in MOH‐M patients. A similar finding was not observed in the TTH group/subgroups.

The type of existing primary headache and the type/quantity of frequently used medications are determinants in the diagnosis and management of MOH (Diener et al. 2016). Migraine as the primary headache increases the risk of MOH development; similarly, the use of combination analgesics, triptans, and opioids increases the risk of MOH development (Hagen et al. 2012; Limmroth et al. 2002). Studies have indicated that low socioeconomic status and low educational level are among the factors that facilitate the development of MOH (Atasoy et al. 2005; Cheung et al. 2015). Depression and anxiety disorders stand out as the most commonly associated conditions with MOH (Diener et al. 2016; Atasoy et al. 2005). Additionally, features resembling dependence, such as tolerance or loss of control over medication use, are observed in these patients (Chen and Wang 2019). The presence of individuals with medication‐overuse or substance abuse in the family is also among the risk factors (Cevoli et al. 2009). In a study evaluating survey results related to addiction, higher dependency scores were reported in patients with MOH (Grande et al. 2009).

### 4.2 Daily Living Activities and Habits

In recent studies, risk factors for MOH have been identified as having a headache frequency of 7–14 days per month initially, being younger than 50 years, experiencing stress, leading a sedentary lifestyle, having a high BMI, smoking, and consuming more than 200 milligrams of caffeine per day (Ertaş and Başağrısı 2018; Chen and Wang 2019; Diener et al. 2019). When the migraine and TTH groups and their subgroups were compared in the present study, no significant difference was found in terms of daily caffeine consumption, smoking, alcohol consumption, and daily physical activity. Additionally, no difference was detected in the Eating Attitudes Test and Eating Disorder Examination Questionnaire scores between the groups. This finding obtained in the present study was not consistent with the limited existing literature. This difference may be attributed to the low number of patients in our study. Furthermore, the factors mentioned could potentially trigger medication overuse, and the chronicization of the primary headache could, in turn, lead to an increase in the patient's current stress, a decrease in daily activities, and elevated anxiety levels, resulting in increased consumption of cigarettes, alcohol, or caffeine. We believe that a comprehensive understanding of this bidirectional relationship requires long‐term studies conducted on large patient groups.

While there is ample data in the literature regarding the association between obesity and migraine, there is less evidence on the relationship between TTH and obesity, and these findings are not as clear as they are in migraine patients (Peres et al. 2005). However, a study conducted in the pediatric age group found that obesity was more prevalent in the patient group with chronic TTH compared to the general population (Pakalnis and Kring 2012). The findings obtained in this study showed that average BMI was not increased in children with MOH or chronic migraine. In the present study, in the TTH group compared to migraine, and in the TTH‐MOH subgroup compared to the MOH‐M subgroup, average BMI was found to be significantly higher. Further investigation of this relationship between obesity and TTH, as well as designing studies to understand the factors involved, will shed more light on this topic.

### 4.3 Disease Perception and Quality of Life

When the SF‐36 findings were evaluated in the subgroups of migraine and TTH patients, significant worsening was found only in bodily pain in the CDH‐M and MOH‐M subgroups, whereas in the CTTH and TTH‐MOH subgroups, significant decline was observed in the subscales of physical role limitation, energy, emotional well‐being, bodily pain, and general health perception. When SF‐36 subscales were compared between MOH‐M and TTH‐MOH groups, only the “physical role limitations” score was found to be statistically significantly higher in the MOH‐M group. This finding indicates that, in terms of the difficulties created by the existing illness in daily or work life, patients with TTH‐MOH experience more challenges compared to patients with MOH‐M. Conversely, the mean score for “psychological causes” in the IPQ was found to be significantly higher in patients with TTH‐MOH compared to patients with MOH‐M. According to this finding, patients with TTH‐MOH attribute their current headache more on psychological causes compared to patients with MOH‐M.

### 4.4 Psychogenic Factors and Psychiatric Comorbidity

Another finding of the present study worth emphasizing is that BAI and BDI scores were significantly higher in the CTTH group compared to the episodic TTH and TTH‐MOH subgroups. For migraine subgroups, anxiety and depression scores were also higher in the CDH‐M group compared to the MOH‐M group; however, this difference was not statistically significant.

We are struggling to explain this finding. The small sample size, as well as the fact that the number of patients in groups with less common diagnoses (e.g., the TTH‐MOH group) is similar to the number of patients in subgroups with more common diagnoses (e.g., episodic migraine), may have introduced a bias. Other potential factors include the variety of medications taken by patients with medication overuse, including anxiolytic drugs. Another possible contributing factor could be the patients' confidence in receiving adequate treatment. These potential factors should be considered in future studies with larger sample sizes to further investigate this issue.

Furthermore, the “consequences” subscale scores of IPQ were highest in the TTH‐MOH group. This finding suggests that the physical, social, financial, and psychological impacts of the illness are lower in TTH‐MOH patients compared to CTTH patients.

One of the most important limitations of the present study was the relatively small sample size, which was partly due to the hospital being designated as a pandemic hospital during Covid‐19 pandemic. During the pandemic, it was observed that patients with episodic headaches had a lower rate of hospital visits. In contrast, patients with chronic headaches were more likely to take the risk and visit the hospital, which may have contributed to the smaller number of episodic headache patients in the study. This, for example, could explain why the number of patients in the TTH‐MOH group was similar to that of the episodic migraine group, potentially influencing the results. Furthermore, we believe that the mandatory restrictions on daily life and social activities during the pandemic may have indirectly influenced the factors we aimed to investigate in the study.

Another important limitation was that the Severity of Dependence Scale could not be used in the study due to the absence of a valid and reliable Turkish version. The fact that episodic headaches can convert to medication‐overuse headache and our inability to examine genetic factors that may affect the prognosis of some patients in the treatment of this headache constituted another limitation of the study. The pandemic posed a major obstacle in reaching out to the family members of patients; therefore, not being able to include the family histories of MOH patients in terms of both MOH and other substance uses was another limitation of the study.

## 5 Conclusion

The experience gained during the study and the obtained data have helped us to gain insights for future studies. First and foremost, when investigating multifactorial medical issues such as the chronicization of episodic headaches and the causes of medication‐overuse headaches, a large sample size for both the case and control groups is necessary. We believe that studies on BMI, an area with few studies in the TTH group, should be planned, as this is an under‐researched field. Taking into account both similar medical histories in family members and risk situations in terms of the factors under investigation, it is essential to include these parameters in the study. Especially in patients with medication overuse, the use of dependency scales and evaluating dependence through face‐to‐face clinical interviews are important. Finally, considering and including genetic factors that may affect both dependence and the treatment process of patients may provide further insights.

## Author Contributions

**Gülcan Taşçatan**: conceptualization, investigation, writing–original draft, methodology, validation, visualization, writing–review and editing, project administration, data curation, resources. **Hüseyin Tuğrul Atasoy**: conceptualization, writing–original draft, methodology, validation, visualization, writing–review and editing, project administration, data curation, supervision, resources. **Esra Aciman Demirel**: conceptualization, writing–original draft, methodology, validation, visualization, writing–review and editing, project administration, supervision, resources. **Vildan Çakır Kardeş**: methodology, writing–review and editing, project administration, resources. **Mustafa Açıkgöz**: visualization, writing–review and editing. **Ulufer Çelebi**: visualization, writing–review and editing. **Bilge Piri Çınar**: writing–original draft, validation, writing–review and editing. **Bilgehan Açıkgöz**: writing–review and editing, formal analysis. **Aynur Özge**: Visualization, writing–review and editing.

### Peer Review

The peer review history for this article is available at https://publons.com/publon/10.1002/brb3.70407

## Data Availability

The data that support the findings of this study are available from the corresponding author upon reasonable request.
