# Correction to “Evaluation of Risk Factors in Patients with Chronic Daily Headache and Medication‐Overuse Headache”

**DOI:** 10.1002/brb3.70503

**Published:** 2025-05-21

Taşçatan, G., H. T. Atasoy, E. A. Demirel, et al. 2025. “Evaluation of Risk Factors in Patients With Chronic Daily Headache and Medication‐Overuse Headache.” *Brain and Behavior* 15: e70407. https://doi.org/10.1002/brb3.70407.

In the originally published version, the affiliation for Gülcan Taşçatan was incorrect. The corrected affiliations appear below. The online version of the article has been corrected accordingly.

Gülcan Taşçatan,^1^ Hüseyin Tuğrul Atasoy,^2^ Esra Acıman Demirel,^2^ Vildan Çakır Kardeş,^3^ Mustafa Açıkgöz,^2^ Ulufer Çelebi,^2^ Bilge Piri Çınar,^4^ Bilgehan Açıkgöz,^5^ Aynur Özge^6^

^1^Zonguldak Alapli State Hospital, Alaplı/Zonguldak, Turkey

^2^Department of Neurology, Zonguldak Bulent Ecevit University Faculty of Medicine, Zonguldak, Turkey

^3^Department of Mental Health and Diseases, Zonguldak Bulent Ecevit University Faculty of Medicine, Zonguldak, Turkey

^4^Department of Neurology, Samsun University Faculty of Medicine, Samsun, Turkey

^5^Department of Public Health, Zonguldak Bulent Ecevit University Faculty of Medicine, Zonguldak, Turkey

^6^Department of Neurology, Mersin University Faculty of Medicine, Yenişehir/Mersin, Turkey

We apologize for the error.

